# Impact of Intravenous Fluid Challenge Infusion Time on Macrocirculation and Endothelial Glycocalyx in Surgical and Critically Ill Patients

**DOI:** 10.1155/2018/8925345

**Published:** 2018-11-01

**Authors:** Jiri Pouska, Vaclav Tegl, David Astapenko, Vladimir Cerny, Christian Lehmann, Jan Benes

**Affiliations:** ^1^Dpt. of Anesthesiology and Intensive Care Medicine, Faculty of Medicine in Plzen, Charles University, Plzen, Czech Republic; ^2^Dpt. of Anesthesiology and Intensive Care Medicine, University Hospital in Plzen, Plzen, Czech Republic; ^3^Biomedical Center, Faculty of Medicine in Plzen, Charles University, Plzen, Czech Republic; ^4^Department of Anaesthesiology and Intensive Care, Charles University in Prague, Faculty of Medicine in Hradec Kralove, Hradec Kralove, Czech Republic; ^5^Faculty of Medicine in Hradec Kralove, Charles University, Hradec Kralove, Czech Republic; ^6^Department of Anaesthesiology, Perioperative Medicine and Intensive Care, J. E. Purkinje University, Masaryk Hospital, Usti nad Labem, Czech Republic; ^7^Centrum for Research and Development, University Hospital Hradec Kralove, Hradec Kralove, Czech Republic; ^8^Department of Anaesthesia, Pain Management and Perioperative Medicine, Dalhousie University, Halifax, NS, Canada

## Abstract

*(i) Purpose.* The fluid challenge (FC) is a well-established test of preload reserve. Only limited data exist in regard to the FC efficacy based on infusion time. Slow administration may be associated with lack of effect based on fluid redistribution and external conditions changes. On the contrary, fast administration may lead to brisk fluid overload and damage to the endothelium and endothelial glycocalyx (EG). The aim of this trial was to compare the FC infusion time on its hemodynamic effects and EG.* (ii) Methods.* Prospective randomized single-center trial of fast (5-10 minutes) versus slow (20-30 minutes) administration of 500ml balanced crystalloid FC in spinal surgery (cohort OR) and septic shock (cohort SEP) patients. Hemodynamic response was assessed using standard monitoring and blood flow measurements; damage to EG was assessed using the perfused boundary region (PBR) via intravital microscopy monitoring in the sublingual region within relevant time points ranging up to 120 minutes.* (iii) Results.* Overall, 66 FCs in 50 surgical and 16 septic patients were assessed. Fluid administration was associated with increase of PBR in general (1.9 (1.8-2.1) vs. 2.0 (1.8-2.2); p= 0.008). These changes were transient in OR cohort whereas they were long-lasting in septic fluid responders. The rate of fluid responsiveness after fast versus slow administration was comparable in global population (15 (47%) vs. 17 (50%); p=0.801) as well as in both cohorts.* (iv) Conclusions.* Fluid challenge administration was associated with increased PBR (and presumable EG volume changes) which normalized within the next 60 minutes in surgical patients but remained impeded in septic fluid responders. The fluid responsiveness rate after fast and slow FC was comparable, but fast administration tended to induce higher, though transient, response in blood pressure.

## 1. Introduction

Fluid resuscitation is considered standard of care in intensive care medicine and the operating room (OR), respectively. Over the years, adverse effects of fluid administration have been increasingly acknowledged [1]; therefore the current view stresses the use of fluid responsiveness prediction and rational use of intravenous infusions [2]. The fluid challenge (FC) is a widely accepted test of preload reserve. However, given the data from the recent FENICE study [3], the FC may differ significantly in several ways (volume, time, type of fluid, variables used for assessment, etc.). Whereas long term effects [4], volume [5], and several other variables have been studied, the rate of administration and its impact on the circulation, and hence test results, remain largely unrevealed. If the administration of crystalloid FC is longer than 10 minutes, the expected time of volume redistribution based on Hahn volume-kinetic studies [6], one can expect to lose some of the FC volume hence making it more prone to false negative assessment. Besides patient's conditions may change dramatically (especially in the OR) debasing the evaluation of fluid response.

Based on the effectivity of mini-FC tests [7] the impact of rapid bolus administration on the central hemodynamic compartment may induce larger increase in venous return and hence test the preload reserve to a larger extent. On the other side, such rush increase in venous return may overcome the reserve in patients on the Frank-Starling curve plateau and may unnecessarily increase the risk of brisk and temporary fluid overload. Hypothetically, such brisk overload may affect the patient's conditions, for instance, by release of natriuretic peptides and endothelial glycocalyx disruption [8]. These conditions may then impact the fluid redistribution and hence final volume effect. In other words, infusion time of FC may significantly alter its macro- and microhemodynamic effects.

Endothelial glycocalyx (EG) is a thin gel-like layer on the outer surface of endothelial cells within the human body. It consists of sugar-based macromolecules such as heparan sulfate or proteoglycan. This structure has been considered as a crucial regulator of endothelial functions such as endothelial permeability or interaction with circulating cells [9]. It is supposed that this layer may get easily harmed by various stimuli and during different disease conditions. The interaction between EG and fluids seems to be bilateral; on one side EG may get disrupted by alterations in circulation volume and pressure and on the other EG is the principal regulator of permeability and fluid extravascular leakage [10, 11]. From this point of view the knowledge of EG behavior during FC and/or fluid therapy is of the upmost importance. However, up until recently only laboratory indirect measures of EG damage (i.e., plasma levels of heparan sulfate or syndecan-1) were available for EG assessment. Monitoring of the sublingual mucosa microcirculation via side-stream dark field (SDF) imaging has been improved over the last decade enabling real-time computation of several characteristics. The perfused boundary region (PBR) has been used by some to inspect the microvascular EG layer thickness hence enabling real-time EG assessment [12, 13]. It describes the extent of penetration of the flowing red blood cells (RBC) in *μ*m into the luminal surface of the EG by measuring the radial motion of RBC away from the central flow towards the endothelial cells. The more the EG is injured, the deeper the RBC penetrate into the glycocalyx and the higher the PBR is.

The aim of this study was to assess the influence of fluid challenge infusion time on macrohemodynamic variables and PBR in surgical and septic patients. Our hypothesis was that faster fluid challenge leads to bigger macrohemodynamic effects but harms EG more than the slower one.

## 2. Materials and Methods

This was a prospective open randomized trial performed at the Department of Anesthesiology and Intensive Care Medicine in Plzen (Charles University Hospital, Czech Republic) in two three-month periods between January and December 2016. The study was approved by the local ethical committee, registered under the ACTRN12618000385246, and financially supported by the Ministry of Health grant no. 15-31881A. Informed consent was obtained from all individual participants (or their legal representatives/next of kin) included in the study.

### 2.1. Patients' Selection

Patients undergoing scheduled operative intervention (cohort OR) or admitted to the ICU for sepsis/septic shock (cohort SEP) in the suspected need for fluid administration/response test based on clinical assessment were recruited into the trial.

The first cohort (OR) consisted of patients scheduled for major spinal surgery under general anesthesia of more than one-hour expected length. Inclusion criteria were age over 18 years, ASA physical status I-III, and signed informed consent. Patients with pathology in the oral cavity (bleeding, neoplasia), previously diagnosed systemic microangiopathy, and atrial fibrillation were excluded from the trial. General anesthesia for the operative procedure was induced using propofol, sufentanil, and atracurium; sevoflurane in O2/Air mixture was used for maintenance. The patients were orotracheally intubated and proned after induction. Ringerfundin (B Braun Melsungen, Germany) at rate of 1ml/kg/hour was administered throughout the procedure. Standard monitoring of noninvasive blood pressure, pulse oximetry, and electrocardiography was commenced; Massimo rainbow plethysmograph with PVI assessment was used in addition to monitor fluid responsiveness. All patients were mechanically ventilated in volume-controlled mode (tidal volume 8 ml/kg of predicted body weight, PEEP 5 cmH_2_O).

The second cohort (SEP) consisted of patients with sepsis or septic shock admitted to the intensive care within 48 hours from ICU admission (mostly within first 24 hours, i.e., during the fluid-optimization phase). Patients younger than 18 years and those with intraoral bleeding and/or diathesis were excluded. Informed consent of the patients was obtained prior to inclusion. In case of patient's diminished consciousness either next of kin or independent physician was asked for consent; patient's agreement was obtained retrospectively if possible. All patients were initially fluid-resuscitated and monitored using the transpulmonary thermodilution (PiCCO2 device, Pulsion-Gettinge, Munich, Germany) in addition to standard ICU monitoring. If needed vasoactive drugs (norepinephrine, dobutamine) were used to reach generally accepted targets of global perfusion parameters (MAP at least 65 mmHg adapted according to individual baseline characteristics and cardiac performance). Analgesia, sedation, and/or mechanical ventilation were used as appropriate.

### 2.2. Randomization and Study Intervention

Patients were randomized by the research staff using the sealed envelope block randomization scheme (https://sealedenvelope.com/) with predefined stratification (OR and SEP). A fluid challenge of 5 ml/kg predicted body weight of crystalloid (Ringerfundin, B Braun Melsungen GmbH) was used as a test of fluid response. Patients were randomly separated into two groups: fast administration (group F; bolus within 5-10min using an infusion cuff pressured at 300mmHg) and slow (group S; 25-30 minutes infusion using an infusion pump at calculated appropriate rate). The study was designed as open so neither blinding of the operator nor outcome assessment was provided.

Based on different time course in the OR and SEP cohort the study time points differed slightly. However, T0 (immediately before FC), T1 (immediately after FC), and T60 (60 minutes after FC) time points measurements were performed in both cohorts. In addition, T20 and T40 (20 and 40 minutes after FC) were made in OR cohort and T120 (120 minutes after FC) was performed in SEP patients.

In case of sudden circulating blood volume change (i.e., blood loss) or need for other fluid administration the monitoring was discontinued and following time points were omitted. Relevant cointerventions within the study period were screened (i.e., change in maintenance infusion rate, administration of other fluid volumes for intravenous medication). However, we have tried to restrict such interfering factors to the minimum by postponing them unless vitally important. The change of vasoactive medication in septic patients (SEP cohort) was allowed during the study period to maintain the blood pressure within predefined range (65-75 mmHg in patients without chronic hypertension and 70-80 in those with chronic hypertension).

### 2.3. Study Outcomes

Primary objective was to assess the influence of balanced crystalloid fluid challenge infusion time on the PBR as a marker of endothelial glycocalyx thickness using the SDF imaging in the sublingual circulation.

Secondary outcomes were to assess its effect on macrocirculation (i.e., rate of positive response) and the temporary differences of the hemodynamic and PBR response in different subgroups (fluid responders=FR, nonresponders= NR, cohort OR and SEP).

### 2.4. Microcirculation and Fluid Response Assessment

The perfused boundary region parameter was used to monitor the possible EG damage induced by FC. This parameter (PBR) was designated to determine the extent of penetration of the flowing red blood cells in capillary to its luminal border by calculating the radial motion of red blood cell away from central flow in capillary. In the situation of EG damage through various pathologic stimuli, this spreading closer to endothelial cells is more obvious and thus the PBR value becomes higher. An intravital real-time microscopy of sublingual circulation by specialized hand-held video microscope (KK camera, Research Technology Limited, Alliance Court, Honiton, UK) was performed at each time point. Acquired data were processed with GlycoCheck software version 1.2.5.7211 (GlycoCheck, Maastricht, Netherlands). The software automatically measures PBR in vessels of diameter from 5 to 25 *μ*m and the resulting number stands for an average of PBR that is corrected for the potential changes in the distribution of vessel diameters. The software identifies all available vessels and places 10 *μ*m long vascular segments along them. Next, a sequence of 40 frames on this spot is recorded with approximately 300 segments in the field. Afterwards, the operator should reposition camera slightly allowing for recording of the next 40-frame sequence. Automated signal quality assessment is performed by the software itself displaying a direct user friendly feedback enabling gathering only valid data. The maximal data-sampling period is 5 minutes making the monitoring less prone to interobserver variability. However, the recording stops automatically when 3000 segments in focus and without movement are acquired; hence under normal conditions much shorter periods (i.e., 1-2 minutes) are needed. Then the software selects segments with sufficient contrast with the background and counts the median RBC column width and its distribution from the intensity profile. From this intensity profile, the perfused diameter of the vessel is calculated by a linear regression analysis. The PBR stands for the distance between RBC column width and perfused diameter according to the equation: (perfused diameter – median RBC column width)/2.

Besides the PBR, automated parameters based on two quality checks, which indirectly align to microvascular perfusion, were assessed in each time point. During first check the so-called “valid vascular segments” are reported (those with more than 60% contrast in vessel segments) making the parameter of valid vessel density. During a second check phase red blood cells (RBC) column is monitored allowing for the calculation of percentage of vascular segments with RBC present in all 40 frames of the monitoring session. Results of the monitoring are available within several minutes after the monitoring period had finished. Detailed description of automatic calculation of PBR, valid and total vascular density, and RBC filling parameters can be found elsewhere [12]. Excellent interobserver validity has been demonstrated by Rovas et al. [14]. In our study, two monitoring procedures were performed (both sides of the sublingual region) and averaged at each time point. JP performed all microcirculatory measurements in the surgical population, whereas JB and VT were responsible for the septic cohort monitoring.

Macrohemodynamic changes were assessed using standard hemodynamic variables based on cohort-defined monitoring. In the OR cohort heart rate, noninvasive blood pressure, pulse pressure, and PVI parameters were used. A drop in PVI of 5% and more between T0 and T1 was measure of a positive fluid response.

In the SEP cohort heart rate, invasive blood pressure (systolic, mean, and pulse pressure values), cardiac output/stroke volume values, stroke volume variation, and thermodilution derived global end-diastolic volume index (GEDI) were assessed. A stroke volume increase of 15% (T1 versus T0) was used to define positive fluid response.

### 2.5. Statistical Analysis

The study was designed as a pilot study, because no baseline data existed regarding both study outcomes (i.e., difference in macrohemodynamic and EG data). A sample of 50 patients in the cohort OR was deemed sufficient to ascertain a 10% difference in the PBR value with alpha error I =0.05 and study power = 0.9. The length of the study limited recruitment into the septic cohort and number of admitted septic patients therefore underwent no prior sample size calculation.

The usual descriptive statistics was used to compare patients between F and S groups; Kolmogorov-Smirnov test was used for assessing normality. Student's t-test (unpaired) or Mann-Whitney rank sum tests were used to compare intergroup differences; the time-based changes within groups were tested using RM ANOVA or RM ANOVA of Ranks (Friedman) appropriately. Chi square test was used to compare frequency distribution. p≤0.05 was deemed statistically significant. MedCalc Statistical Software version 18.2.1 (MedCalc Software bvba, Ostend, Belgium; http://www.medcalc.org; 2018) was used for statistical analysis.

## 3. Results

In total 66 patients have been included and 66 FCs have been studied during the run of the study; there were no drop-offs. In the OR cohort 50 fluid challenges were performed in 50 patients equally randomized to receive either fast (group F-OR: 25 FCs) or slow (group S-OR: 25 FCs) FC administration. In the SEP cohort in 16 patients, 16 fluid challenges were performed, randomized to fast (group F-SEP: 7 FCs) and slow (group S-SEP: 9 FCs) FC administration; the median time from sepsis onset (or hospital admission for sepsis) and intervention was 16 (7-33) hours. There were no dropouts; the flow of participants through the trial is displayed in e-Figure 1. Baseline demographic and disease characteristics are given in Table 1; no significance has been observed in any of screened parameters.

The perfused boundary region was comparable at the baseline between F and S group (1.8 (1.72-2.05) vs. 1.89 (1.76-2.08); p= 0.176). The PBR increased after FC in general (1.88 (1.76-2.08) vs. 1.95 (1.79-2.20); p= 0.008) and in both groups in parallel (T1 in F group 1.91 (1.75-2.13) vs. 1.96 (1.85-2.26) in S; p=0.256). The PBR remained increased throughout the next course in both groups (T60 values in F were 1.89 (1.77-2.08) vs. 2.02 (1.72-2.16) in S; p=0.576); see Table 2 for more details. No differences in other microcirculatory parameters (i.e., number of valid vessel density and RBC filling) were observed (see ESM Table 1).

Based on the responsiveness criteria in global 32 FCs (49%) were assessed as positive: 24 (48%) in the OR and eight (50%) in the SEP cohort. No difference in the FC outcome was identified between fast and slow administration neither in global population nor in any of the cohorts (Table 3).

No differences in screened hemodynamic variables were observed at the baseline between F and S groups in any of the cohorts studied (Table 4). However, the blood pressure increase induced directly by fluid infusion occurred in the F group only (78 ± 11 mmHg in T0 vs. 86 ± 12 mmHg in T1; p=0.004) leading to higher MAP in the F group at T1 (86 ± 12 mmHg vs. 77 ± 12 mmHg; p=0.003). Interestingly, this increase equalized in the 60 minutes time point (84 ± 11 mmHg in the F group vs. 84 ± 12 mmHg in the S group; p=0.086). The dynamic variation of stroke volume or plethysmography variability index (PVI) decreased in both groups after the FC and tended to increase back after 60 minutes, but these changes went in parallel in both groups. Pulse pressure (PP) increased after the fluid challenge in the F group only (37 (32-56) mmHg in T0 vs. 43 (36-61) mmHg in T1; p=0.019), but decreased afterwards in the T60 time point. On the contrary, in the S group the PP increase was prolonged reaching its peak at T60. No major differences were observed in heart rate and other hemodynamic parameters; see Table 4 for more details.

### 3.1. Differences between Anesthetized and Septic Patients

**Figure 1:**
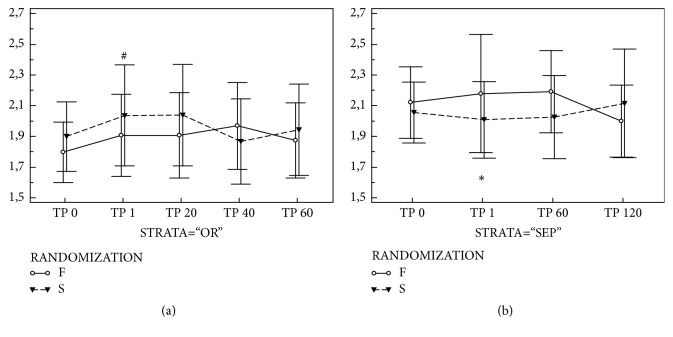
**Perfused boundary region changes induced by fluid administration. **Legend: Panel (a) PBR changes in surgical patients (Cohort OR); Panel (b) PBR changes in patients with sepsis/septic shock (Cohort SEP). *Abbreviations: Group F (white): fast administration; Group S (grey): slow administration; PBR: perfused boundary region; TP 0, 1, 20, 40, 60, 120: measurement point immediately before, immediately after, and at 20, 40, 60, and 120 minutes after fluid challenge; #: significant difference against baseline in the slow group; ∗: significant difference between fast and slow group*.

In septic patients, the PBR values were significantly higher (2.08 (1.90-2.22) in SEP vs. 1.81 (1.73-2.00) in OR cohort; p<0.001) at the baseline. In the S-OR subgroup there was an increase of the PBR after FC with later return to initial values (Table 2, Figure 1(a)). In the F-OR subgroup the PBR values increased in T1 as well, but without reaching statistical significance. However, no intergroup differences were observed between F-OR and S-OR groups at any time point. In the septic patients both the PBR remained stable without intergroup (i.e., F-SEP vs. S-SEP) or temporary changes (Figure 1(b)). No important variations were observed in number of valid vessel density and RBC filling among study groups within the study period (ESM Table 1).

As mentioned previously in global FC response, there were no differences among any subgroup studied (i.e., OR, SEP; Table 3). The pressure response to FC was apparently faster in the F-OR group than S-OR (87 ± 14 mmHg in T1 vs. 75 ± 13 mmHg; p=0.003), but equalized within the next 20 minutes (91 ± 13 mmHg in T20 vs. 88 ± 11 mmHg; p=0.52) and remained comparable throughout the next time points (T40 and T60) (ESM Table 1). A similar pattern was observed in the pulse pressure. The T1 values were higher in the F-OR subgroup (37 (34-43) mmHg vs. 32 (28-41) mmHg in S-OR; p=0,048), but equalized during T20, T40, and T60 time points (ESM Table 1). A nonsignificant drop in the value of PVI was observed in both F-OR and S-OR subgroups, without any intergroup differences.

In the SEP cohort there were differences neither in MAP at the T1 (77 ± 10 mmHg in F-SEP vs. 82 ± 9 mmHg in S-SEP; p=0.404), nor at any other time point afterwards (T60 and T120; ESM Table 1) between F-SEP and S-SEP subgroups. However, the dose of norepinephrine dropped in F-SEP group in T1 and remained decreased until T60 presuming a better pressure response. No important changes in neither the pulse pressure, stroke volume variation, and flow (stroke volume (SV) or cardiac index (CI)) nor the volumetric (global end-diastolic volume index (GEDI) and extravascular lung water index (ELWI)) parameters were observed between F-SEP and S-SEP groups (ESM Table 1).

### 3.2. Analysis Based on Fluid Response

**Figure 2:**
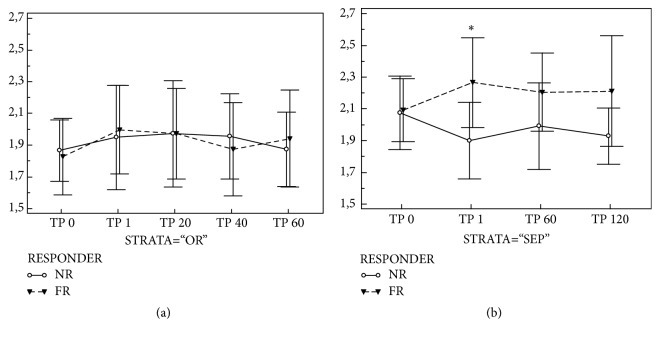
**Perfused boundary region changes based on fluid challenge response. **Legend: Panel (a) PBR changes in surgical patients (Cohort OR); Panel (b) PBR changes in patients with sepsis/septic shock (Cohort SEP). *Abbreviations: Responder FR (white): patients with positive hemodynamic response to fluid challenge; NON-Responder NR (grey): patients with negative hemodynamic response to fluid challenge; PBR: perfused boundary region; TP 0, 1, 20, 40, 60, 120: measurement point immediately before, immediately after, and at 20, 40, 60, and 120 minutes after fluid challenge; ∗: significant difference between responders and non-reposnders.*

The PBR values were comparable at the baseline between FR and NR patients. In FR patients the PBR increased after FC (1.85 (1.74 – 2.08) vs. 2.10 (1.86 – 2.78); p=0.014), but remained stable in NR patients. This PBR increase occurred in fluid responders of both OR (1.76 (1.73-2.02) vs. 1.96 (1.79-2.26); p=0.016) and SEP cohort (1.96 (1.85-2.18) vs. 2.28 (2.04-2.52); p=0.039), but in SEP fluid responders the PBR not only increased against baseline value, but rose significantly even in comparison with fluid nonresponders (see Figure 2, panels (a) and (b)).

The fluid nonresponders (NR) tended to have lower baseline value of the PVI in the OR cohort (10 % (6-16) vs. 19 % (13-22) in the FR; p= 0.001) and SVV (9 % (6-12) vs. 10 % (3-15) in NR; p=0.87). The initial values of MAP, PP, HR, SV, CI, GEDI, and ELWI were all comparable between fluid responders and nonresponders at the baseline; for full results, see ESM Table 2. In septic fluid responders the norepinephrine dose was lower in FR already at the baseline (0.23 (0.09-0.45) vs. 0.5 (0.39-1.03) in NR; p= 0.049) and dropped further after FC (0.23 (0.09-0.46) *μ*g/kg/min in T0 to 0.19 (0.05-0.32) in T1) and remained decreased until T120. No such change occurred in nonresponding patients. On the contrary, the central venous pressure value rose in nonresponding septic patients only (ESM Table 2).

## 4. Discussion

In our trial, fluid administration in form of fluid challenge increased the PBR value independently of the infusion time. However, there may be a weak signal that septic fluid responders seem to be more affected. Secondly, the 5-10 minutes' or 20-30 minutes' infusion time of fluid challenge seems not to play an important role regarding rate of positive fluid response. However, a transient increase of mean arterial pressure and/or decrease in norepinephrine needs was more pronounced after fast administration.

To ascertain the impact of different infusion time on the microcirculation we have used the perfused boundary region parameter. Based on previous observations the PBR may be linked to the endothelial glycocalyx thickness [12, 13] and hence point to its disruption by the fluids administration. This unwanted effect of fluid boluses has been demonstrated using serum markers of EG damage by several authors [8, 15, 16]. In our trial, the PBR increased after almost any fluid administration. In patients with presumably intact endothelial surface layer (i.e., OR cohort), the increase after slow administration reached statistical significance against baseline. However, in the F-OR the increase went in parallel, making this finding difficult to address the FC infusion time. Also, the PBR returned to initial values within 60 minutes in both groups. Such fast changes in the PBR parameter, especially the return to initial value, actually point towards a significant EG disruption. Possibly other forms of volume change within the structure (i.e., shrinkage or dilution) may occur explaining this effect.

On the contrary, in the septic patients the PBR pointed towards existing EG damage induced by sepsis and/or previous fluid boluses. Higher PBR values after FC were observed in septic fluid responders than nonresponders, but infusion time does not seem to play any role. Whether this PBR increase is due to recruitment of capillaries and hence local ischemia/reperfusion remains undetermined. The slightly lower density of valid microvessels and trend towards lower RBC filling support the hypothesis of capillary recruitment associated with hemodilution. Another important fact is that, unlike in OR patients in whom the PBR returned to preinfusion values within 60 minutes, in septic fluid responders the PBR remained increased for the next 2 hours. Therefore, another explanation could be that the increased systemic flow and precapillary pressure induces higher shear stress [17] to the already damaged endothelia promoting existing damage. In a recent retrospective evaluation Puskarich et al [15] have demonstrated that higher volumes of resuscitation fluids in septic patients were associated with intubation and increased syndecan-1 levels, observations pointing to the promoted EG damage by fluids in septic patients. Hypothetically, those patients who do respond to initial fluid bolus tend to receive more fluids and based on our observations they are actually more endangered by the EG shedding. However, our findings in septic population are based on very small patient cohort; hence these findings have to be proven on much larger patient population.

The impact of fluids on EG has been studied previously. Mentioned studies by Chappell et al. [8], Powell et al. [16], and Puskarich et al. [15] have demonstrated that fluid administration may induce damage to the EG. Most recently a Slovenian group has demonstrated that intraoperative liberal fluid administration may be associated with EG disruption [18]. Biochemical EG shedding markers (i.e., syndecan-1, hyaluronan, etc.) were used in these studies to demonstrate the unwanted effect of fluids administration. Such markers were not available for our study for technical reasons (unavailability of such analysis in our institution). This may be regarded as an important drawback. However, EG shedding markers are not only endothelium specific. Divergent inflammatory conditions (especially in the SEP cohort) would probably interfere with their levels independently of fluid administration [19, 20]. Also the kinetics of these markers seem to be rather prolonged: in the Slovenian study [18] an increase of syndecan-1 level increased after the surgery, but remained high for the next six hours. Besides, to which extent the plasma increase of such shedding markers mirrors a real decrease in EG thickness or is a sigh of increase turnover has never been demonstrated. Described prolonged kinetics and need for off-site analysis preclude the use of such markers as bed-side EG-disruption marker. For this reason, we deem the PBR to be more clinically relevant as a possible marker of endothelial damage. However, amount of data regarding the PBR is still too low to allow us for separation of clinically relevant changes. In our study the highest increase observed among septic responders was 8% which lies on the borderline of the coefficient of variation measured on our population (8%) or mentioned by other authors (less than 10%) [21]. The normal values in population have also not been evaluated yet. In the Netherlands Epidemiology of Obesity study the mean values in the total population (42% lean, 42% overweight, and 16% obese) were 2.14+/-0.25 [12]. In the GlycoNurse study [14] the median value and interquartile range of PBR in patients admitted into the emergency department for various reasons including sepsis (40%) or acute heart failure (16%) were 2.41 (2.26-2.61). Values measured in our population (both “healthy” surgical and septic patients) were much lower. Interestingly, in recently published Russian population study [21] the PBR values corresponded much more with our own measurements; median and interquartile values were 1.9 (1.75-2.04) with values above 2 pointing towards increased cardiovascular risk. To which extent these differences in absolute values are affected by different SDF camera device (KK Research technology Ltd., UK, in ours and Russian study [21] vs. MicroVision Medical Inc., Wallingford, PA, in the Dutch study [12]) version of monitoring software (mostly not given) or population is to be further elucidated.

Fluid challenge is a well-established test of preload reserve. Interestingly, the number of fluid responders and nonresponders is stable throughout different studies and patients' populations. The fact that rate of fluid challenge should affect its outcome has been postulated already by Weil and Vincent in the original TROL acronym (Type-Rate-Objectives-Limits of safety) [22]. In general, the 20-30 minutes seems to be the right choice. Based on the FENICE trial data the median time of FC administration of 500 ml (median volume) was 24 minutes [3]. However, a wide variety existed. In a recent meta-analysis concerning FC technique [23] the effect of FC was similar among studies with administration lasting <15 and 15-29 minutes (59.2% vs. 57.7% of fluid responders) whereas in those with prolonged infusion, i.e., >30 minutes, the positive response was less frequent (49.9%; p=0.045 for <15min vs. >30 min). However, these data are an indirect comparison only and based on studies included into the meta-analysis (table 1 in Toscani et al. [23]) in the <15 minutes group there was a high predominance of operating theatre studies (70% out of 27 trials) whereas both the other groups (15-29 minutes and >30 minutes) were ICU studies only. Hence, the observed difference may be attributed to the population under study as well. In another recent meta-analysis concerning the FC, none of screened variables (infusion time, sepsis, hypotension, oliguria, and use of colloids) correlated with the FC hemodynamic outcome [24]. Our results do not contradict these observations supporting the fact that FC administered within 30 minutes does not lead to different outcomes in terms of responsive positivity rate. One important fact remains largely underrecognized by a portion of these studies: the FC's volume mostly administered is 500 ml, which is administered in a dedicated time period making the administration rate fixed and comparable between different subjects. However, based on the body size the circulating blood volume may significantly differ among subjects making the actual blood volume expansion and hence hemodynamic response different in comparable time points (i.e., after 15 or 30 minutes). Therefore, in our study, we have used a FC's volume based on patients predicted body weight. This led to different administration rates, but fixed circulating blood volume expansions in the study time points.

The longevity and delayed response to the FC were a secondary measure of our trial. In this regard, both short and prolonged infusions seem to have similar characteristics. In the fast infusion groups, a more pronounced pressure response was observed, but within the next 20-60 minutes, the blood pressure was equal in both groups. Unlike in the study by Aya et al. [4] the effect of crystalloid FC lasted till the end of observation (60 minutes in surgical and 120 minutes in septic patients). Different volume of FC may partially explain this observed difference; in Aya et al.'s study a 5 minutes' bolus FC of 250 ml was used; this corresponds with a relative volume of 3ml/kg (whereas 5ml/kg was used in our study). The same group (Aya et al.) has clearly demonstrated that at least 4ml/kg has to be used in the 5 minutes' FC to induce a clear-cut hemodynamic response [5]. The delayed, but comparable, hemodynamic effect of slow vs. fast infusion further supports the data observed by Hahn et al. [25] and Ukor et al. [26]. In multiple studies, Hahn et al. postulated based on hemoglobin dilution measurements that the expansion of circulating volume after crystalloid administration (especially after prolonged infusion) is far bigger that theorized. They have also demonstrated a significant context sensitivity of the phenomenon [6]. Ukor et al. compared an infusion of one-liter saline to 6 healthy volunteers over 30 minutes and 2 hours [26]. The 2 hours' infusion produced more stable hemodynamic response as measured via noninvasive technology. Hemoglobin values were not consistently monitored in our patients, but the PiCCO cardiac output device demonstrated a stable profile in hemodynamic in the later course especially after slow FC administration. However, one has to bear in mind that studying one simple intervention (as fluid administration) in a dynamic system (as any clinical situation) for a prolonged times is extremely difficult. Not only does redistribution occur which may be studied on relatively stable conditions of volunteers, but also other influences may contribute to the results (in our case surgery and anesthesia depth in surgical patients, change in vasoactive medications in septic patients). Still, the answer may be of upmost clinical relevance, namely, in the case of fluids administration whose adverse effects coupled with accumulation are nowadays well known. In our trial, we have attempted to minimize the confounders throughout the study period to the minimum: by choosing a relatively stable surgical procedure or by postponing all other medical interventions not vitally indicated (literally, only vasoactive drugs were adapted based on predefined protocol to reach generally accepted MAP targets).

Our study has several important limitations, which need to be accounted for. Firstly, in the OR cohort fluid responsiveness was assessed based on clinically available parameter usually coupled with positive response to fluid administration (decrease in PVI). Use of advanced hemodynamic monitoring would possibly define the secondary outcome more precisely. The reason to use the readily available noninvasive monitoring tools was based on the ethical request not to increase the invasivity of the monitoring used and noninvasive hemodynamic monitor was not technically available at the time of the study. Therefore, we have used parameter, which is routinely available and possibly acceptable for definition of positive fluid response. In addition, the conditions in spinal surgery are rather specific (prone position, no exposure of the body cavities, limited blood loss) limiting the generalizability of our results into other populations (i.e., intra-abdominal, vascular, thoracic surgery, etc.). Secondly, the number of septic patients recruited into the study is low and could influence observed results. As mentioned in the methods section the inclusion of these patients was limited by the time of device availability and we have recruited the whole cohort of septic patients admitted to the ICU throughout the study period. Besides, the negative finding on the influence of fluid challenge infusion time goes in line with previous observations as reviewed by Toscani et al. [23] and Messina et al. [24].

In conclusion, in our trial fluid challenge administration was associated with increased perfused boundary region (and presumable volume changes of the endothelial glycocalyx) which normalized within the next 60 minutes in surgical patients but remained impeded in septic fluid responders.

The fluid responsiveness rate after fast (5-10 minutes) and slow (20-30 minutes) fluid challenge administration was comparable in our patients. Fast administration tended to induce higher, but transient, response in blood pressure.

## Figures and Tables

**Table 1:** Baseline characteristics.

	Group F			Group S		
	All	Cohort OR	Cohort SEP	All	Cohort OR	Cohort SEP
	(N=32)	(N=25)	(N=7)	(N=34)	(N=25)	(N=9)
Age (years)	60.1 ± 14.7	59.5 ± 12.9	61.1 ± 18.1	60 ± 16.6	61.7 ± 15.2	56.7 ± 19.3

Sex (Female/male)	18/14	14/11	4/3	18/16	15/10	3/6

Height (cm)	167.8 ± 12.5	169.3 ± 11.2	165 ± 26	167.3 ± 11.1	168 ± 8.8	165.9 ± 26.1

Weight (kg)	88.8 ± 26.3	85.6 ± 17.3	95.7 ± 39.3	87.2 ± 26.2	82.9 ± 15.1	95.8 ± 39.8

Predicted body weight (kg)	64.0 ± 11.1	64.0 ± 11.3	64.2 ± 11.5	64.0 ± 9.8	62.3 ± 8.9	65.9 ± 11.2

Fluid challenge infusion rate (ml/min)	46 (39-54)	44 (39-54)	47 (40-52)	11 (9-12)	10 (9-12)	11 (10-13)

**Chronic conditions**						

ASA grade (1/2/3)	-	5/9/11	-	-	5/13/6	-

APACHE II score	-	-	26.6 ± 7.2	-	-	27.2 ± 9.6

SOFA score	-	-	10 (7.3 – 14.3)	-	-	8 (7.8 – 12.3)

Hypertension (no.)	21	15	3	17	12	4

Smoking (no.)	10	6	3	9	4	2

Diabetes (no.)	10	9	1	6	5	1

**Operative procedure**						

Laminectomy	-	9	-	-	6	-

Discectomy	-	5	-	-	14	-

Fixation	-	11	-	-	5	-

Intraoperative blood loss (ml)	-	175	-	-	300	-
		(100 – 300)			(188-400)	

**Sepsis origin and nature**						

Lungs	-	-	2	-	-	5

Abdomen	-	-	5	-	-	2

Bloodstream	-	-	0	-	-	1

Orofacial	-	-	0	-	-	1

Vasopressor use (no (%))	-	-	6 (86%)	-	-	8 (89%)

Noradrenalin dose (*μ*g/kg/min)	-	-	0.46	-	-	0.38
			(0.13-0.50)			(0.24-0.71)

Septic shock	-	-	4 (57%)	-	-	8 (89%)

**Baseline biochemistry**						

Hematocrit (%)	39.6 ± 6.3	42.6 ± 3.3	33.6 ± 6.6	39.8 ± 5.9	42.5 ± 3.4	34.2 ± 6.2

Sodium (mmol/l)	140.9 ± 3.6	140.7 ± 2.7	141 ± 5.0	140.7 ± 4.2	140.4 ± 2.9	141.3 ± 6.3

Protein (g/l)	63.7 ± 9.8	68.4 ± 7.4	55.6 ± 19.3	66.3 ± 9.7	71.4 ± 11.3	55.6 ± 8.2

Creatinine (umol/l)	103.6 ± 67.4	75 ± 19.2	160.7 ± 91.4	95.5 ± 59.5	71.9 ± 4.1	145.7 ± 85.2

C-reactive protein (mg/l)	-	-	266.6 ± 30.0	-	-	265.2 ± 29.9

Procalcitonin (ug/l)	-	-	16.0 ± 8.5	-	-	16.1 ± 8.5

Serum lactate (mmol/l)	-	-	2.18 ± 1.27	-	-	2.66 ± 0.73

Data are displayed as mean ± standard deviation, median (25th-75th percentile), or number (proportion).

*Abbreviations: APACHE II: Acute Physiology and Chronic Health Evaluation score-second version; ASA: American Society of Anesthesiology physiological status; SOFA: Sequential Organ Failure Assessment.*

**Table 2:** Perfused boundary region values.

Variable	group	timepoint	Group Fast		Group Slow		p value	Non-RESPONDER		RESPONDER		p value
			(n=32)		(n=34)			(n=27)		(n=39)		
Perfused boundary region	all	T0	1.80	1.72 to 2.05	1.89	1.76 to 2.08	0.176	1.89	1.78 to 2.04	**1.86**	1.76 to 2.10	0.667
		T1	1.91	1.75 to 2.13	1.96	1.85 to 2.26	0.256	**1.89**	**1.78 to 2.10**	**2.10** **∗**	**1.86 to 2.28**	**0.043**
		T60	1.89	1.77 to 2.08	2.02	1.72 to 2.16	0.576	1.88	1.74 to 2.13	2.04	1.79 to 2.24	0.146
		p value	0.095		0.224			0.521		0.014		
	OR	T0	1.77	1.70 to 1.94	**1.86**	1.76 to 2.04	0.118	1.85	1.73 to 1.95	1.76	1.73 to 2.02	0.336
		T1	1.90	1.77 to 2.08	**1.96** **∗**	1.79 to 2.27	0.171	1.88	1.78 to 2.12	1.96	1.79 to 2.26	0.286
		T20	1.88	1.71 to 2.09	2.04	1.72 to 2.21	0.165	1.98	1.72 to 2.15	1.99	1.73 to 2.14	0.985
		T40	1.99	1.74 to 2.17	1.89	1.62 to 2.05	0.263	1.94	1.74 to 2.15	1.87	1.64 to 2.04	0.346
		T60	1.81	1.74 to 2.00	1.88	1.71 to 2.16	0.584	1.83	1.70 to 1.99	1.90	1.72 to 2.12	0.404
		p value	0.093		**0.034**			0.078		0.154		
	SEP	T0	2.12	1.96 to 2.30	2.02	1.89 to 2.22	0.560	1.97	1.75 to 2.16	1.96	1.85 to 2.18	0.857
		T1	2.10	1.78 to 2.31	1.96	1.90 to 2.08	0.427	**1.99**	**1.78 to 2.07**	**2.28**	**2.04 to 2.52**	**0.027**
		T60	2.01	1.91 to 2.25	2.15	1.84 to 2.17	0.791	2.07	1.85 to 2.17	2.19	2.05 to 2.33	0.115
		T120	1.87	1.77 to 2.22	2.01	1.91 to 2.29	0.418	1.91	1.79 to 2.01	2.05	2.03 to 2.47	0.056
		p value	0.212		0.867			0.376		0.290		

Values are displayed as median (interquartile range). ^*∗*^Significant difference towards baseline (Friedman test), intergroup significant difference marked in bold letters. Abbreviations: Group Fast: fast administration; Group Slow: slow administration; OR: operative cohort; SEP: patients with sepsis/septic shock.

**Table 3:** Fluid challenge outcome.

Population	Fluid responsive				p (chí sqr)
	YES		NO		
	Group F	Group S	Group F	Group S	
Global	15	17	17	17	0.801
(N=66)					

Cohort OR	11	13	14	12	0.575
(N=50)					

Cohort SEP	4	4	3	5	0.626
(N=16)					

Abbreviations: Group F: fast administration; Group S: slow administration; Cohort OR: surgical patients; Cohort SEP: patients with sepsis/septic shock.

**Table 4:** Macrohemodynamic changes induced by fluid challenge in the whole population.

Variable	Time-point	Group F	Group S	P value (between groups)
		(N=32)	(N=34)	
MAP (mmHg)	T0	77 ± 11	78 ± 11	0.800
	T1	**85 ± 13** **∗**	**78 ± 12**	**0.024**
	T60	**85 ± 11** **∗**	**85 ± 12** **∗**	0.986
	p value	**0.027**	**0.025**	

Pulse pressure (mmHg)	T0	37 (30-43)	35 (30-55)	0.893
	T1	40 (35-45)	39 (30-51)	0.338
	T60	38 (31-48)	**40 (31-61)** **∗**	0.496
	p value	0.205	**0.017**	

Heart rate (per minute)	T0	69 (60-79)	70 (63-81)	0.603
	T1	64 (59-75)	68 (58-82)	0.812
	T60	68 (63-87)	74 (65-88)	0.481
		0.174	0.209	

Plethysmography Variability index (%) (OR cohort)	T0	13 (7-20)	15 (12-21)	0.281
	T1	10 (7-11)	10 (7-14)	0.276
	T60	13 (8-17)	11 (10-15)	0.879
	p value	0.086	0.087	

Stroke volume variation (%) (SEP cohort)	T0	**12 (10-19)**	**5 (2-9)**	**0.016**
	T1	6 (6-11)	6 (4-10)	0.568
	T60	4 (4-14)	**7 (3-12)** **∗**	0.936
	p value	0.621	**0.013**	

Stroke volume index (ml/m^2^) (SEP cohort)	T0	33 ± 14	40 ± 20	0.515
	T1	32 ± 14	44 ± 20	0.320
	T60	33 ± 13	44 ± 16	0.281
	p value	0.750	0.563	

Values are displayed as either median (interquartile range) or mean ± standard deviation; *∗*: significant difference towards baseline (RM ANOVA or Friedman test), significance marked in bold letters.

*Abbreviations: Group F: fast administration; Group S: slow administration; MAP: mean arterial pressure; PVI: plethysmography variability index; SVV: stroke volume variation; PBR: perfused boundary region; SEP cohort: patients with sepsis/septic shock.*

## Data Availability

The raw data used to support the findings of this study are available from the corresponding author upon request.

## Supplementary Materials

Supplementary Materials The ESM consists of two results tables displaying the detailed results of each variable at each time point in the general population and each cohort (i.e., surgical and septic patients). Table 1 concerns the separation based on intervention, i.e., groups separated according to fast or slow administration of fluid challenge. Table 2 compares the groups based on fluid challenge results, meaning based on positive or negative fluid response. In addition, the CONSORT flow chart and reference table were added to the ESM based on the reviewers' comments.
