# Supplementary material for: Impact of Intravenous Fluid Challenge Infusion Time on Macrocirculation and Endothelial Glycocalyx in Surgical and Critically Ill Patients
Source: Biomed Res Int. 2018 Nov 1;2018:8925345. doi: 10.1155/2018/8925345 (PMC6241356; doi:10.1155/2018/8925345)
Supplement: Supplementary Materials — The ESM consists of two results tables displaying the detailed results of each variable at each time point in the general population and each cohort (i.e., surgical and septic patients). Table 1 concerns the separation based on intervention, i.e., groups separated according to fast or slow administration of fluid challenge. Table 2 compares the groups based on fluid challenge results, meaning based on positive or negative fluid response. In addition, the CONSORT flow chart and reference table were added to the ESM based on the reviewers' comments. [file 8925345.f1.docx]

ESM Table 1 – Analysis of subgroups

| Variable | Cohort | timepoint | Group Fast (n=32) | | Group Slow (n=34) | | p value |
| --- | --- | --- | --- | --- | --- | --- | --- |
| Perfused  boundary  region | all | T0 | 1.795 | 1.720 to 2.050 | 1.890 | 1.760 to 2.080 | 0.1757 |
|  |  | T1 | 1.910 | 1.750 to 2.133 | 1.958 | 1.850 to 2.260 | 0.2562 |
|  |  | T60 | 1.888 | 1.770 to 2.076 | 2.021 | 1.722 to 2.160 | 0.5764 |
|  |  | p value | 0.09544 |  | 0.22402 |  |  |
|  | OR | T0 | 1.770 | 1.700 to 1.935 | **1.860** | 1.760 to 2.043 | 0.1183 |
|  |  | T1 | 1.900 | 1.765 to 2.083 | **1.960*** | 1.790 to 2.268 | 0.1713 |
|  |  | T20 | 1.882 | 1.710 to 2.090 | 2.040 | 1.722 to 2.207 | 0.1653 |
|  |  | T40 | 1.992 | 1.737 to 2.165 | 1.887 | 1.621 to 2.053 | 0.2627 |
|  |  | T60 | 1.811 | 1.744 to 1.996 | 1.883 | 1.709 to 2.155 | 0.5839 |
|  |  | p value | 0.093 |  | **0.034** |  |  |
|  | SEP | T0 | 2.120 | 1.961 to 2.295 | 2.020 | 1.888 to 2.220 | 0.5604 |
|  |  | T1 | 2.100 | 1.784 to 2.306 | 1.955 | 1.903 to 2.079 | 0.4273 |
|  |  | T60 | 2.010 | 1.910 to 2.246 | 2.150 | 1.843 to 2.173 | 0.7913 |
|  |  | T120 | 1.870 | 1.773 to 2.216 | 2.005 | 1.905 to 2.288 | 0.4179 |
|  |  | p value | 0.21190 |  | 0.86703 |  |  |
| Valid vessel density | all | T0 | 308.078 | 80.1910 | 280.221 | 64.8931 | 0.1247 |
|  |  | T1 | 297.266 | 98.3004 | 279.176 | 79.7819 | 0.4135 |
|  |  | T60 | 288.607 | 77.2809 | 292.741 | 89.1167 | 0.8525 |
|  |  | p value | 0.51561 |  | 0.85216 |  |  |
|  | OR | T0 | **327.760** | **76.4626** | **282.840** | **62.6237** | **0.0276** |
|  |  | T1 | 312.800 | 99.8912 | 280.320 | 83.7092 | 0.2188 |
|  |  | T20 | 324.640 | 79.7396 | 285.160 | 85.7796 | 0.0984 |
|  |  | T40 | 308.667 | 60.7902 | 309.440 | 92.5383 | 0.9727 |
|  |  | T60 | 303.952 | 77.9618 | 292.750 | 77.4120 | 0.6470 |
|  |  | p value | 0.16835 |  | 0.78582 |  |  |
|  | SEP | T0 | 237.786 | 49.5991 | 272.944 | 74.3280 | 0.3005 |
|  |  | T1 | 241.786 | 73.5923 | 276.000 | 72.2435 | 0.3670 |
|  |  | T60 | 242.571 | 57.6082 | 292.722 | 116.4635 | 0.3164 |
|  |  | T120 | 263.357 | 84.5920 | 265.625 | 65.7070 | 0.9543 |
|  |  | p value | 0.68407 |  | 0.85216 |  |  |
| RBC filling (%) | all | T0 | 73.752 | 5.4948 | 72.454 | 5.9501 | 0.3618 |
|  |  | T1 | 72.384 | 6.9871 | 71.351 | 5.3420 | 0.5008 |
|  |  | T60 | 72.784 | 4.1630 | 71.593 | 6.8515 | 0.4332 |
|  |  | p value | 0.90166 |  | 0.08755 |  |  |
|  | OR | T0 | 75.168 | 3.9148 | 73.068 | 6.0005 | 0.1493 |
|  |  | T1 | 73.384 | 7.3283 | 71.916 | 5.2928 | 0.4208 |
|  |  | T20 | 73.720 | 5.7443 | 70.504 | 5.9408 | 0.0575 |
|  |  | T40 | 72.988 | 5.7578 | 73.764 | 5.5651 | 0.6334 |
|  |  | T60 | 73.238 | 3.6538 | 72.600 | 6.8554 | 0.7101 |
|  |  | p value | 0.18326 |  | 0.43721 |  |  |
|  | SEP | T0 | 68.693 | 7.5151 | 70.750 | 5.7947 | 0.5454 |
|  |  | T1 | 68.814 | 4.2882 | 69.783 | 5.4692 | 0.7062 |
|  |  | T60 | 71.421 | 5.5315 | 69.356 | 6.6721 | 0.5198 |
|  |  | T120 | 70.850 | 8.4187 | 69.188 | 6.0176 | 0.6639 |
|  |  | p value | 0.87511 |  | 0.19235 |  |  |
| Mean arterial pressure  (mmHg) | all | T0 | **77.094** | 10.8133 | 77.765 | 10.6118 | 0.8000 |
|  |  | T1 | **85.094*** | **13.1624** | **77.971** | **11.8896** | **0.0241** |
|  |  | T60 | **84.778*** | **10.9381** | **84.724*** | 12.3314 | 0.9864 |
|  |  | p value | **0.02720** |  | **0.02523** |  |  |
|  | OR | T0 | **77.000** | 11.1617 | **76.400** | 11.0076 | 0.8490 |
|  |  | T1 | **86.840*** | **13.8072** | **74.800** | **13.1053** | **0.0027** |
|  |  | T20 | **90.667*** | 12.9857 | **88.440*** | 11.2117 | 0.5232 |
|  |  | T40 | **92.435*** | 14.4320 | **96.334*** | 10.4084 | 0.2859 |
|  |  | T60 | 87.100 | 11.1351 | 87.250 | 12.1823 | 0.9678 |
|  |  | p value | **0.002** |  | **<0.001** |  |  |
|  | SEP | T0 | 77.429 | 10.2771 | 81.556 | 8.8898 | 0.4036 |
|  |  | T1 | 78.857 | 8.6300 | 83.111 | 8.2681 | 0.3334 |
|  |  | T60 | 78.143 | 7.5151 | 79.111 | 11.3297 | 0.8486 |
|  |  | T120 | 78.000 | 5.7155 | 79.250 | 11.2472 | 0.7953 |
|  |  | p value | 0.84512 |  | 0.39891 |  |  |
| Norepinephrine dose  (μg/kg/min) | SEP | T0 | **0.455** | 0.132 to 0.500 | 0.380 | 0.237 to 0.705 | 0.8137 |
|  |  | T1 | **0.315*** | 0.100 to 0.500 | 0.380 | 0.188 to 0.705 | 0.7683 |
|  |  | T60 | **0.375*** | 0.100 to 0.560 | 0.420 | 0.188 to 0.678 | 0.9530 |
|  |  | T120 | 0.375 | 0.132 to 0.630 | 0.425 | 0.224 to 0.630 | 0.7469 |
|  |  | p value | 0.00709 |  | 0.11811 |  |  |
| Pulse  pressure (mmHg) | all | T0 | 37.000 | 29.500 to 43.000 | **34.500** | 30.000 to 55.000 | 0.8927 |
|  |  | T1 | 40.000 | 35.250 to 44.750 | 38.500 | 30.000 to 51.000 | 0.3375 |
|  |  | T60 | 38.000 | 31.000 to 48.250 | **40.000*** | 31.000 to 60.500 | 0.4960 |
|  |  | p value | 0.20478 |  | 0.01651 |  |  |
|  | OR | T0 | 34.000 | 26.750 to 37.000 | 30.000 | 27.750 to 37.000 | 0.4600 |
|  |  | T1 | **37.000** | **34.000 to 43.000** | **32.000** | **28.000 to 41.000** | **0.0477** |
|  |  | T20 | 34.500 | 28.500 to 38.000 | 33.000 | 30.000 to 37.250 | 0.6890 |
|  |  | T40 | 33.000 | 27.250 to 37.750 | 35.000 | 29.250 to 40.000 | 0.4957 |
|  |  | T60 | 34.000 | 29.000 to 40.000 | 33.500 | 30.000 to 40.500 | 0.7865 |
|  |  | p value | 0.093 |  | 0.073 |  |  |
|  | SEP | T0 | 62.000 | 42.500 to 72.250 | 62.000 | 56.000 to 84.750 | 0.6338 |
|  |  | T1 | 61.000 | 47.250 to 68.250 | 67.000 | 60.750 to 81.750 | 0.3146 |
|  |  | T60 | 68.000 | 49.500 to 70.000 | 64.000 | 61.000 to 70.750 | 0.8738 |
|  |  | T120 | 64.000 | 51.500 to 70.500 | 67.000 | 58.500 to 75.000 | 0.4519 |
|  |  | p value | 0.99803 |  | 0.30434 |  |  |
| Plethysmography variability index)  (%) | OR | T0 | 13.00 | 7.000 to 20.000 | 15.000 | 11.500 to 21.250 | 0.2807 |
|  |  | T1 | 9.500 | 7.000 to 11.000 | 10.000 | 7.000 to 14.000 | 0.2758 |
|  |  | T20 | 11.000 | 9.000 to 16.000 | 10.500 | 8.500 to 16.000 | 1.0000 |
|  |  | T40 | 12.500 | 9.000 to 14.000 | 14.000 | 8.000 to 17.000 | 0.3178 |
|  |  | T60 | 12.500 | 8.000 to 17.000 | 11.000 | 10.000 to 15.000 | 0.8786 |
|  |  | p value | 0.086 |  | 0.087 |  |  |
| Stroke volume variation  (%) | SEP | T0 | **12.000** | **10.000 to 19.000** | **4.500** | **2.000 to 9.000** | **0.0163** |
|  |  | T1 | 6.000 | 6.000 to 11.000 | 6.000 | 4.000 to 10.000 | 0.5683 |
|  |  | T60 | 4.000 | 4.000 to 14.000 | **6.500*** | 3.000 to 12.000 | 0.9361 |
|  |  | T120 | 5.000 | 3.000 to 14.000 | 5.000 | 3.000 to 8.000 | 0.8089 |
|  |  | p value | 0.62090 |  | 0.01278 |  |  |
| Heart rate  (beats/min) | all | T0 | 68.500 | 60.000 to 79.000 | 70.000 | 63.000 to 81.000 | 0.6033 |
|  |  | T1 | 63.500 | 58.500 to 74.500 | 68.000 | 58.000 to 82.000 | 0.8123 |
|  |  | T60 | 68.000 | 63.250 to 87.000 | 74.000 | 64.750 to 88.000 | 0.4807 |
|  |  | p value | 0.17420 |  | 0.20906 |  |  |
|  | OR | T0 | 65.000 | 59.250 to 72.500 | 68.000 | 61.000 to 75.500 | 0.4319 |
|  |  | T1 | 60.000 | 56.000 to 70.250 | 63.000 | 53.750 to 69.250 | 0.8843 |
|  |  | T20 | 60.000 | 54.750 to 70.000 | 63.000 | 55.750 to 80.000 | 0.3222 |
|  |  | T40 | 64.500 | 57.500 to 69.500 | 63.000 | 56.000 to 82.250 | 0.4592 |
|  |  | T60 | 65.500 | 59.000 to 71.500 | 71.000 | 63.500 to 75.500 | 0.2501 |
|  |  | p value | 0.057 |  | 0.073 |  |  |
|  | SEP | T0 | 116.000 | 105.250 to 125.500 | 100.000 | 79.500 to 103.500 | 0.0719 |
|  |  | T1 | 114.000 | 105.250 to 124.500 | 98.000 | 84.000 to 107.000 | 0.1530 |
|  |  | T60 | 111.000 | 97.750 to 123.750 | 93.000 | 83.750 to 106.000 | 0.1009 |
|  |  | T120 | 109.000 | 103.750 to 121.250 | 94.500 | 84.000 to 108.000 | 0.1182 |
|  |  | p value | 0.12737 |  | 0.85768 |  |  |
| Cardiac index (l/min/m^2^) | SEP | T0 | 4.200 | 2.325 to 5.300 | 3.400 | 2.475 to 4.200 | 0.6015 |
|  |  | T1 | 4.400 | 2.325 to 4.815 | 3.200 | 2.875 to 4.325 | 0.9168 |
|  |  | T60 | 3.800 | 2.475 to 5.100 | 3.500 | 3.175 to 4.675 | 0.7540 |
|  |  | T120 | 4.200 | 2.400 to 4.700 | 3.800 | 3.450 to 5.100 | 0.8065 |
|  |  | p value | 0.69117 |  | 0.55236 |  |  |
| Stroke volume (ml) | SEP | T0 | 32.800 | 14.0606 | 40.200 | 19.8419 | 0.5154 |
|  |  | T1 | 32.400 | 13.8311 | 43.800 | 19.6901 | 0.3204 |
|  |  | T60 | 33.000 | 13.1529 | 43.600 | 15.7258 | 0.2810 |
|  |  | T120 | 32.600 | 12.7789 | 52.000 | 16.6733 | 0.0877 |
|  |  | p value | 0.74970 |  | 0.56262 |  |  |
| Central venous pressure (mmHg) | SEP | T0 | 8.000 | 6.250 to 11.500 | 11.500 | 8.500 to 12.500 | 0.4175 |
|  |  | T1 | 10.000 | 6.250 to 14.250 | 11.500 | 8.500 to 13.000 | 0.6025 |
|  |  | T60 | 10.000 | 5.250 to 12.500 | 11.500 | 8.000 to 12.500 | 0.5625 |
|  |  | T120 | 10.000 | 5.000 to 12.000 | 12.000 | 10.250 to 13.000 | 0.3056 |
|  |  | p value | 0.46657 |  | 0.57270 |  |  |
| Systemic Vascular resistance index  (dyne.s.cm^-5^.m^-2^) | SEP | T0 | 1390.000 | 1095.000 to 2577.500 | 1599.000 | 1496.250 to 1867.250 | 0.7540 |
|  |  | T1 | 1306.000 | 1076.000 to 2473.500 | 1876.000 | 1363.500 to 2054.000 | 0.9168 |
|  |  | T60 | 1565.000 | 1118.500 to 2494.250 | 1391.000 | 1120.500 to 1937.750 | 0.6015 |
|  |  | T120 | 1309.000 | 1166.000 to 2418.500 | 1321.500 | 1136.000 to 1554.000 | 0.8065 |
|  |  | p value | 0.81756 |  | 0.21719 |  |  |
| Global end-diastolic index (ml/m^2^) | SEP | T0 | **819.500** | 770.500 to 1189.500 | 733.000 | 682.500 to 829.500 | 0.2207 |
|  |  | T1 | **871.500*** | 839.500 to 1232.500 | 751.000 | 594.000 to 894.000 | 0.2864 |
|  |  | T60 | **880.500*** | 839.000 to 1202.500 | 736.000 | 587.000 to 864.000 | 0.1356 |
|  |  | T120 | **861.500*** | 819.000 to 1193.500 | 725.000 | 590.000 to 847.000 | 0.1356 |
|  |  | p value | 0.00044 |  | 0.05542 |  |  |
| Extra-vascular lung water index (ml/kg) | SEP | T0 | 13.500 | 10.500 to 18.500 | 15.000 | 9.250 to 27.500 | 0.7133 |
|  |  | T1 | 12.500 | 10.000 to 19.500 | 25.500 | 8.000 to 37.000 | 0.5224 |
|  |  | T60 | 12.500 | 9.500 to 18.500 | 24.000 | 7.000 to 38.000 | 0.5940 |
|  |  | T120 | 12.000 | 9.500 to 18.500 | 25.000 | 8.000 to 37.000 | 0.5224 |
|  |  | p value | 0.36152 |  | 0.84606 |  |  |
| Legend: The data are displayed as mean ± standard deviation or median (interquartile range); p values in last column correspond with intergroup comparison using Mann-Whitney or independent samples t-test. p values in line bellow timepoints in each stratum correspond to the RM ANOVA or Friedmann test p value. asterix (*) marks timepoints significantly different from the baseline.  Abbreviations: Cohort OR – surgical patients; SEP – septic patients; Timepoints: T0 – immediately before fluid challenge. T1 – immediately after fluid challenge. T20..120 – 20..120 minutes after fluid challenge; | | | | | | | |

| ESM Table 2 – Analysis based on fluid response | | | | | | | |
| --- | --- | --- | --- | --- | --- | --- | --- |
| Variable | Cohort | timepoint | Non-RESPONDER  (n=27) | | RESPONDER (n=39) | | p value |
| Perfused  boundary  region | all | T0 | 1.890 | 1.780 to 2.035 | **1.860** | 1.760 to 2.103 | 0.6672 |
|  |  | T1 | **1.890** | **1.780 to 2.095** | **2.095*** | **1.860 to 2.275** | **0.0433** |
|  |  | T60 | 1.880 | 1.736 to 2.128 | 2.043 | 1.791 to 2.237 | 0.1463 |
|  |  | p value | 0.52142 |  | 0.01422 |  |  |
|  | OR | T0 | 1.850 | 1.730 to 1.950 | 1.760 | 1.730 to 2.015 | 0.3360 |
|  |  | T1 | 1.875 | 1.780 to 2.120 | 1.960 | 1.785 to 2.255 | 0.2855 |
|  |  | T20 | 1.984 | 1.722 to 2.149 | 1.992 | 1.725 to 2.142 | 0.9845 |
|  |  | T40 | 1.938 | 1.739 to 2.146 | 1.874 | 1.644 to 2.039 | 0.3464 |
|  |  | T60 | 1.834 | 1.701 to 1.988 | 1.900 | 1.719 to 2.122 | 0.4039 |
|  |  | p value | 0.07795 |  | 0.15352 |  |  |
|  | SEP | T0 | 1.972 | 1.750 to 2.160 | 1.955 | 1.850 to 2.180 | 0.8571 |
|  |  | T1 | **1.988** | **1.775 to 2.068** | **2.278** | **2.038 to 2.515** | **0.0274** |
|  |  | T60 | 2.065 | 1.845 to 2.170 | 2.185 | 2.050 to 2.331 | 0.1152 |
|  |  | T120 | 1.905 | 1.788 to 2.010 | 2.050 | 2.027 to 2.471 | 0.0562 |
|  |  | p value | 0.37599 |  | 0.28971 |  |  |
| Valid vessel density | all | T0 | 297.676 | 75.7684 | 289.531 | 71.9692 | 0.6562 |
|  |  | T1 | 296.206 | 95.1717 | 279.172 | 82.5619 | 0.4414 |
|  |  | T60 | 297.310 | 85.0041 | 283.875 | 81.4169 | 0.5450 |
|  |  | p value | 0.96724 |  | 0.52638 |  |  |
|  | OR | T0 | 308.692 | 74.0674 | 301.625 | 72.7974 | 0.7354 |
|  |  | T1 | 300.538 | 99.0855 | 292.250 | 87.1112 | 0.7556 |
|  |  | T20 | 314.769 | 90.2026 | 294.208 | 78.0167 | 0.3947 |
|  |  | T40 | 314.077 | 68.6772 | 303.391 | 88.2186 | 0.6363 |
|  |  | T60 | 301.333 | 73.1993 | 295.500 | 82.4605 | 0.8117 |
|  |  | p value | 0.61119 |  | 0.76512 |  |  |
|  | SEP | T0 | 261.875 | 74.5361 | 253.250 | 59.3272 | 0.8016 |
|  |  | T1 | 282.125 | 85.6737 | 239.937 | 53.9288 | 0.2582 |
|  |  | T60 | 286.750 | 115.8355 | 254.812 | 75.8951 | 0.5248 |
|  |  | T120 | 255.375 | 85.5811 | 275.071 | 58.3934 | 0.6170 |
|  |  | p value | 0.98737 |  | 0.59505 |  |  |
| RBC filling (%) | all | T0 | 73.057 | 6.1734 | 73.111 | 5.3107 | 0.9700 |
|  |  | T1 | 72.496 | 6.5179 | 71.169 | 5.7971 | 0.3866 |
|  |  | T60 | 73.393 | 5.0734 | 70.920 | 6.0696 | 0.1003 |
|  |  | p value | 0.81003 |  | 0.14718 |  |  |
|  | OR | T0 | 74.362 | 5.2822 | 73.854 | 5.0490 | 0.7304 |
|  |  | T1 | 73.035 | 6.9075 | 72.233 | 5.8507 | 0.6614 |
|  |  | T20 | 73.208 | 6.9700 | 70.925 | 4.6095 | 0.1822 |
|  |  | T40 | 73.831 | 4.0651 | 72.878 | 7.0355 | 0.5589 |
|  |  | T60 | 74.362 | 4.1226 | 71.420 | 6.2220 | 0.0807 |
|  |  | p value | 0.61171 |  | 0.19821 |  |  |
|  | SEP | T0 | 68.819 | 7.2917 | 70.881 | 5.7934 | 0.5411 |
|  |  | T1 | 70.744 | 5.0284 | 67.975 | 4.5619 | 0.2680 |
|  |  | T60 | 70.850 | 6.6477 | 69.669 | 5.8749 | 0.7121 |
|  |  | T120 | 71.694 | 5.8109 | 67.986 | 8.1788 | 0.3250 |
|  |  | p value | 0.93045 |  | 0.08827 |  |  |
| Mean arterial pressure  (mmHg) | all | T0 | 79.118 | 10.4845 | 75.656 | 10.6608 | 0.1884 |
|  |  | T1 | 82.500 | 12.6736 | 80.281 | 13.3083 | 0.4903 |
|  |  | T60 | 84.250 | 11.3190 | 85.250 | 12.0112 | 0.7497 |
|  |  | p value | 0.25130 |  | 0.14984 |  |  |
|  | OR | **T0** | **79.000** | 11.1319 | **74.208** | 10.4631 | 0.1242 |
|  |  | T1 | 83.077 | 13.3414 | 79.750 | 15.0629 | 0.4117 |
|  |  | T20 | **90.974*** | 11.8042 | **88.027*** | 12.3456 | 0.3974 |
|  |  | T40 | **91.987*** | 10.1513 | **97.160*** | 14.4138 | 0.1548 |
|  |  | T60 | 87.400 | 9.6431 | **86.950*** | 13.3908 | 0.9036 |
|  |  | p value | 0.00029 |  | 0.00003 |  |  |
|  | SEP | T0 | 79.5000 | 8.6850 | 80.0000 | 10.7171 | 0.9198 |
|  |  | T1 | 80.6250 | 10.7828 | 81.8750 | 5.9146 | 0.7780 |
|  |  | T60 | 76.3750 | 11.9396 | 81.0000 | 6.3920 | 0.3505 |
|  |  | T120 | 77.3750 | 11.4385 | 80.1429 | 4.8795 | 0.5636 |
|  |  | p value | 0.76792 |  | 0.97960 |  |  |
| Norepinephrine dose  (μg/kg/min) | SEP | T0 | **0.500** | **0.390 to 1.025** | **0.231** | **0.091 to 0.455** | **0.0491** |
|  |  | T1 | **0.500** | **0.390 to 1.025** | **0.185*** | **0.050 to 0.315** | **0.0151** |
|  |  | T60 | **0.560** | **0.420 to 0.898** | **0.185*** | **0.050 to 0.375** | **0.0279** |
|  |  | T120 | **0.590** | **0.423 to 0.908** | **0.190*** | **0.144 to 0.403** | **0.0409** |
|  |  | p value | 0.87969 |  | 0.00015 |  |  |
| Pulse  pressure (mmHg) | all | T0 | 34.000 | 28.000 to 41.000 | 37.000 | 31.000 to 55.500 | 0.0976 |
|  |  | T1 | **37.000** | **30.000 to 44.000** | **41.000** | **35.000 to 59.250** | **0.0292** |
|  |  | T60 | 35.000 | 29.000 to 49.500 | 40.000 | 33.500 to 60.000 | 0.0739 |
|  |  | p value | 0.17985 |  | 0.49336 |  |  |
|  | OR | T0 | 30.000 | 26.000 to 36.000 | 35.500 | 30.000 to 38.500 | 0.0719 |
|  |  | T1 | **30.500** | **29.000 to 39.000** | **39.000** | **34.000 to 43.250** | **0.0129** |
|  |  | T20 | 34.000 | 26.750 to 38.500 | 33.500 | 30.500 to 37.500 | 0.8414 |
|  |  | T40 | 33.000 | 26.750 to 37.250 | 35.000 | 30.000 to 40.000 | 0.2352 |
|  |  | T60 | **31.000** | **27.500 to 36.500** | **37.500** | **31.000 to 40.500** | **0.0335** |
|  |  | p value | 0.90467 |  | 0.39411 |  |  |
|  | SEP | T0 | 60.000 | 40.500 to 73.500 | 66.000 | 56.000 to 81.500 | 0.4309 |
|  |  | T1 | 63.500 | 43.500 to 75.000 | 68.000 | 60.500 to 76.000 | 0.3720 |
|  |  | T60 | 61.000 | 48.000 to 65.500 | 69.500 | 65.000 to 73.000 | 0.0661 |
|  |  | T120 | 58.500 | 50.000 to 69.500 | 69.000 | 64.500 to 76.000 | 0.1649 |
|  |  | p value | 0.25707 |  | 0.95081 |  |  |
| Plethysmography variability index)  (%) | OR | T0 | **10.000** | 6.000 to 15.750 | **19.000** | 13.000 to 22.000 | 0.0011 |
|  |  | T1 | **11.500** | **8.000 to 18.000** | **8.500*** | **6.500 to 11.000** | **0.0233** |
|  |  | T20 | 11.000 | 9.000 to 16.750 | **11.000*** | 9.000 to 15.000 | 0.6757 |
|  |  | T40 | **13.000*** | 8.500 to 15.750 | **13.000*** | 8.000 to 16.000 | 0.7135 |
|  |  | T60 | **14.000*** | **11.000 to 17.000** | **10.500*** | **9.500 to 12.500** | **0.0407** |
|  |  | p value | 0.02991 |  | <0.00001 |  |  |
| Stroke volume variation  (%) | SEP | T0 | 9.000 | 6.0000 to 12.2500 | 10.000 | 2.7500 to 15.0000 | 0.8710 |
|  |  | T1 | 6.000 | 6.0000 to 10.7500 | 6.000 | 3.7500 to 7.2500 | 0.4085 |
|  |  | T60 | 12.000 | 4.7500 to 14.0000 | 4.000 | 2.7500 to 4.5000 | 0.0509 |
|  |  | T120 | 8.000 | 3.7500 to 16.2500 | 3.000 | 3.0000 to 4.7500 | 0.1907 |
|  |  | p value | 0.15194 |  | 0.87079 |  |  |
| Heart rate  (beats/min) | all | T0 | 69.000 | 62.000 to 79.000 | **70.000** | 61.000 to 85.000 | 0.8524 |
|  |  | T1 | 68.000 | 60.000 to 82.000 | **64.500*** | 56.000 to 81.000 | 0.6077 |
|  |  | T60 | 71.000 | 64.000 to 87.000 | 74.000 | 64.000 to 90.000 | 0.8763 |
|  |  | p value | 0.82554 |  | 0.01237 |  |  |
|  | OR | T0 | 65.500 | 60.000 to 75.000 | **67.000** | 58.500 to 71.000 | 0.8460 |
|  |  | T1 | 63.000 | 59.000 to 70.000 | **59.500*** | **53.500 to 68.500** | 0.2599 |
|  |  | T20 | 63.000 | 55.000 to 72.000 | 62.000 | 54.500 to 74.000 | 0.8995 |
|  |  | T40 | 65.500 | 56.000 to 77.000 | 62.000 | 58.000 to 73.500 | 0.9043 |
|  |  | T60 | 68.500 | 60.500 to 74.500 | 66.500 | 61.500 to 75.000 | 0.8924 |
|  |  | p value | 0.38509 |  | 0.03478 |  |  |
|  | SEP | T0 | 94.5000 | 77.5000 to 132.0000 | 105.0000 | 101.5000 to 111.0000 | 0.6744 |
|  |  | T1 | 96.5000 | 77.0000 to 131.0000 | 105.5000 | 99.0000 to 110.5000 | 0.9581 |
|  |  | T60 | 98.0000 | 85.0000 to 129.5000 | 104.5000 | 94.0000 to 110.0000 | 0.8336 |
|  |  | T120 | 102.0000 | 89.0000 to 128.5000 | 106.0000 | 94.7500 to 109.0000 | 0.8170 |
|  |  | p value | 0.80381 |  | 0.71791 |  |  |
| Cardiac index (l/min/m^2^) | SEP | T0 | 3.650 | 2.6000 to 4.5500 | 3.400 | 2.5000 to 5.1000 | 0.8312 |
|  |  | T1 | 3.500 | 2.5000 to 4.2100 | 3.650 | 2.8000 to 5.4000 | 0.6698 |
|  |  | T60 | 3.400 | 2.5500 to 4.3000 | 4.0500 | 2.8000 to 5.1000 | 0.4555 |
|  |  | T120 | 3.450 | 2.5500 to 4.1000 | 4.200 | 3.6500 to 5.3000 | 0.2207 |
|  |  | p value | 0.89877 |  | 0.82135 |  |  |
| Stroke volume (ml) | SEP | T0 | 35.7500 | 18.0993 | 37.0000 | 17.4126 | 0.9154 |
|  |  | T1 | 33.2500 | 14.9304 | 41.3333 | 19.0543 | 0.4975 |
|  |  | T60 | 34.000 | 14.5144 | 41.1667 | 15.5360 | 0.4849 |
|  |  | T120 | 33.5000 | 14.0594 | 47.4000 | 18.0361 | 0.2482 |
|  |  | p value | 0.91529 |  | 0.3325 |  |  |
| Central venous pressure (mmHg) | SEP | T0 | **12.000** | 10.5000 to 12.7500 | 7.500 | 5.5000 to 11.0000 | 0.0638 |
|  |  | T1 | **12.000*** | 11.2500 to 15.7500 | 10.000 | 5.5000 to 12.0000 | 0.0641 |
|  |  | T60 | 12.000 | 10.000 to 13.000 | 8.5000 | 5.0000 to 11.5000 | 0.1321 |
|  |  | T120 | **11.000*** | 10.0000 to 12.0000 | 12.000 | 5.000 to 13.000 | 0.9489 |
|  |  | p value | 0.00175 |  | 0.78259 |  |  |
| Systemic Vascular resistance index  (dyne.s.cm^-5^.m^-2^) | SEP | T0 | 1630.000 | 1192.5000 to 2134.5000 | 1494.5000 | 1323.0000 to 2351.0000 | 1.0000 |
|  |  | T1 | 1788.0000 | 1183.5000 to 2260.5000 | 1591.0000 | 1140.0000 to 2041.0000 | 0.8312 |
|  |  | T60 | 1607.000 | 1044.0000 to 2202.5000 | 1412.0000 | 1139.0000 to 2282.0000 | 0.8312 |
|  |  | T120 | 1549.500 | 1141.0000 to 2201.5000 | 1309.0000 | 1163.5000 to 1582.2500 | 0.4624 |
|  |  | p value | 0.92194 |  | 0.42622 |  |  |
| Global end-diastolic index (ml/m^2^) | SEP | T0 | 745.5000 | 650.0000 to 1146.0000 | **817.0000** | 757.0000 to 864.0000 | 0.4624 |
|  |  | T1 | 737.0000 | 628.0000 to 1200.5000 | **871.5000*** | 840.0000 to 894.0000 | 0.5224 |
|  |  | T60 | 723.0000 | 614.5000 to 1161.0000 | **872.0000*** | 824.0000 to 881.0000 | 0.3938 |
|  |  | T120 | 710.0000 | 614.0000 to 1151.0000 | 851.5000 | 812.0000 to 867.0000 | 0.5224 |
|  |  | p value | 0.15555 |  | 0.00260 |  |  |
| Extra-vascular lung water index (ml/kg) | SEP | T0 | 18.5000 | 11.0000 to 30.0000 | 12.0000 | 9.7500 to 17.2500 | 0.5403 |
|  |  | T1 | 19.5000 | 11.0000 to 30.5000 | 13.0000 | 10.0000 to 35.0000 | 1.000 |
|  |  | T60 | 18.5000 | 11.0000 to 30.0000 | 12.5000 | 9.0000 to 33.0000 | 0.7491 |
|  |  | T120 | 18.5000 | 11.0000 to 30.0000 | 12.5000 | 9.0000 to 35.0000 | 0.9151 |
|  |  | p value | 0.99390 |  | 0.25527 |  |  |
| Legend: The data are displayed as mean ± standard deviation or median (interquartile range); p values in last column correspond with intergroup comparison using Mann-Whitney or independent samples t-test. p values in line bellow timepoints in each stratum correspond to the RM ANOVA or Friedmann test p value. asterix (*) marks timepoints significantly different from the baseline.  Abbreviations: Cohort OR – surgical patients; SEP – septic patients; Timepoints: T0 – immediately before fluid challenge. T1 – immediately after fluid challenge. T20..120 – 20..120 minutes after fluid challenge; | | | | | | | |

e-Figure 1 – CONSORT flow chart of the patients through the trial


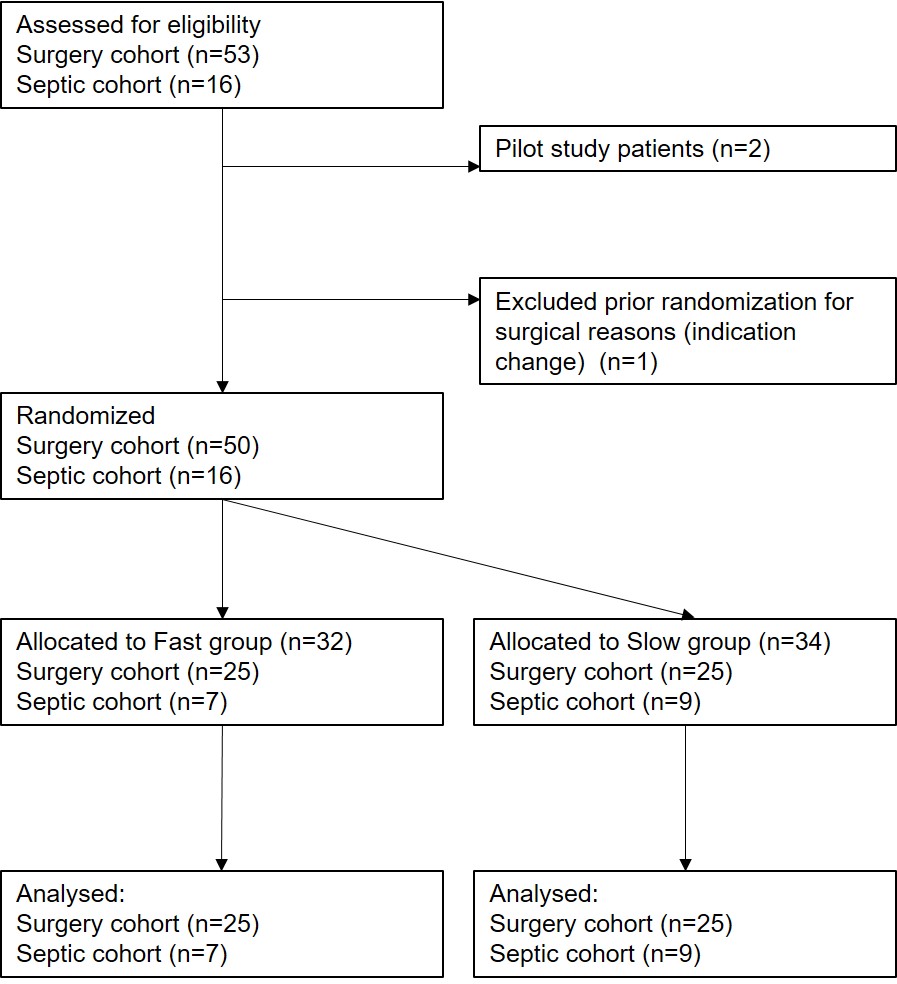


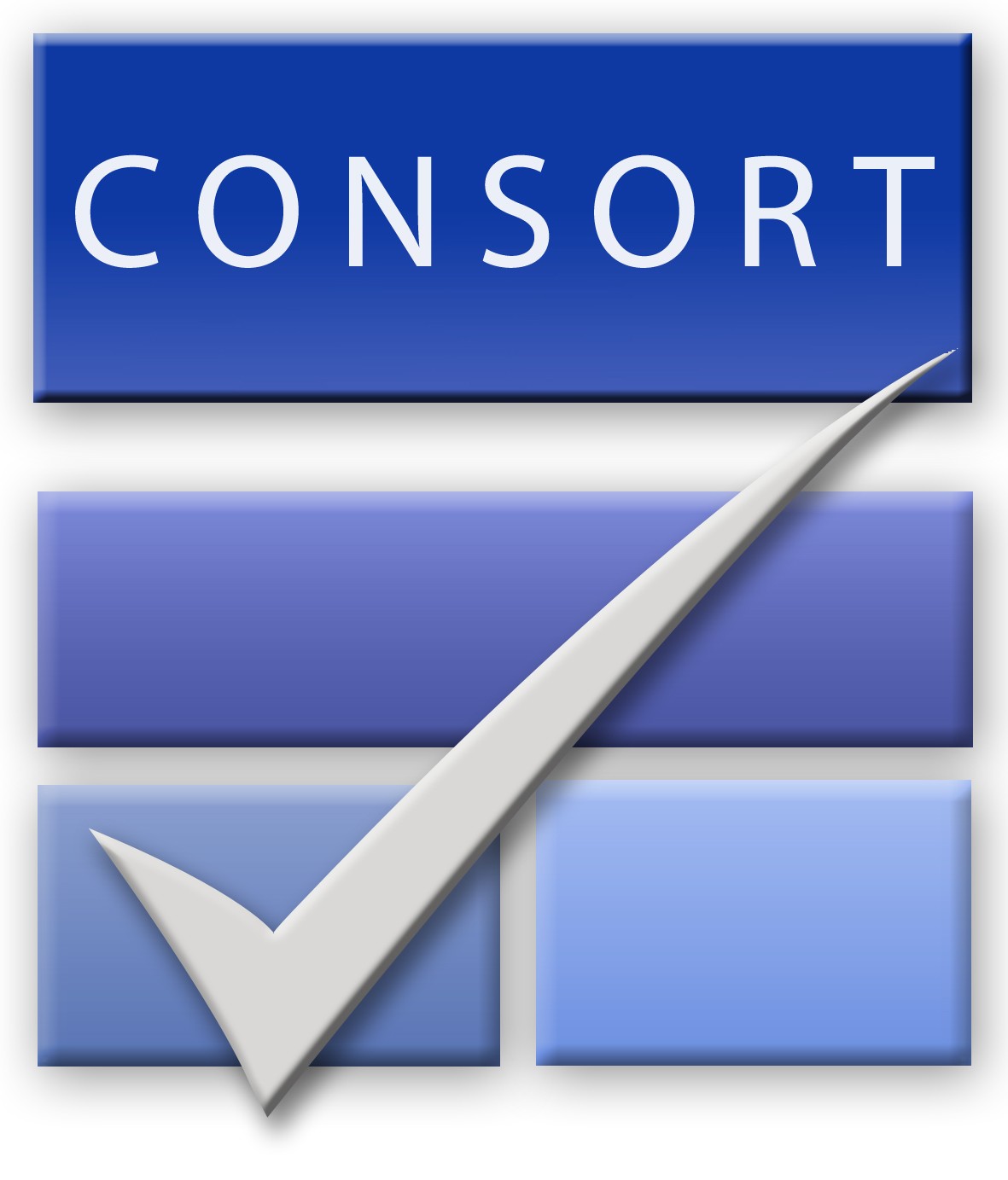
CONSORT 2010 checklist of information to include when reporting a randomised trial*

| Section/Topic | Item No | Checklist item | Reported on page No |
| --- | --- | --- | --- |
| Title and abstract | | | |
|  | 1a | Identification as a randomised trial in the title | No |
|  | 1b | Structured summary of trial design, methods, results, and conclusions (for specific guidance see CONSORT for abstracts) | 3 |
| Introduction | | | |
| Background and objectives | 2a | Scientific background and explanation of rationale | 4 |
|  | 2b | Specific objectives or hypotheses | 4 |
| Methods | | | |
| Trial design | 3a | Description of trial design (such as parallel, factorial) including allocation ratio | 5 |
|  | 3b | Important changes to methods after trial commencement (such as eligibility criteria), with reasons | N/A |
| Participants | 4a | Eligibility criteria for participants | 5 |
|  | 4b | Settings and locations where the data were collected | 5-6 |
| Interventions | 5 | The interventions for each group with sufficient details to allow replication, including how and when they were actually administered | 5 |
| Outcomes | 6a | Completely defined pre-specified primary and secondary outcome measures, including how and when they were assessed | 6-7 |
|  | 6b | Any changes to trial outcomes after the trial commenced, with reasons | N/A |
| Sample size | 7a | How sample size was determined | 7 |
|  | 7b | When applicable, explanation of any interim analyses and stopping guidelines | N/A |
| Randomisation: |  |  |  |
| Sequence generation | 8a | Method used to generate the random allocation sequence | 5 |
|  | 8b | Type of randomisation; details of any restriction (such as blocking and block size) | 5 |
| Allocation concealment mechanism | 9 | Mechanism used to implement the random allocation sequence (such as sequentially numbered containers), describing any steps taken to conceal the sequence until interventions were assigned | 5 |
| Implementation | 10 | Who generated the random allocation sequence, who enrolled participants, and who assigned participants to interventions | 5 |
| Blinding | 11a | If done, who was blinded after assignment to interventions (for example, participants, care providers, those assessing outcomes) and how | 5 |
|  | 11b | If relevant, description of the similarity of interventions | 5 |
| Statistical methods | 12a | Statistical methods used to compare groups for primary and secondary outcomes | 6-7 |
|  | 12b | Methods for additional analyses, such as subgroup analyses and adjusted analyses | 6-7 |
| Results | | | |
| Participant flow (a diagram is strongly recommended) | 13a | For each group, the numbers of participants who were randomly assigned, received intended treatment, and were analysed for the primary outcome | 8, e-Figure 1 |
|  | 13b | For each group, losses and exclusions after randomisation, together with reasons | 8, e-Figure 1 |
| Recruitment | 14a | Dates defining the periods of recruitment and follow-up | 5 |
|  | 14b | Why the trial ended or was stopped | N/A |
| Baseline data | 15 | A table showing baseline demographic and clinical characteristics for each group | Table 1 |
| Numbers analysed | 16 | For each group, number of participants (denominator) included in each analysis and whether the analysis was by original assigned groups | Tables 1-4 + ESM Table 1 and 2 |
| Outcomes and estimation | 17a | For each primary and secondary outcome, results for each group, and the estimated effect size and its precision (such as 95% confidence interval) | 8-9, Tables 2-4 + ESM Tables 1,2 |
|  | 17b | For binary outcomes, presentation of both absolute and relative effect sizes is recommended | 8-9, Tables 2-4 + ESM Tables 1,2 |
| Ancillary analyses | 18 | Results of any other analyses performed, including subgroup analyses and adjusted analyses, distinguishing pre-specified from exploratory | Figures 1+2, + ESM Tables 1,2 |
| Harms | 19 | All important harms or unintended effects in each group (for specific guidance see CONSORT for harms) | N/A |
| Discussion | | | |
| Limitations | 20 | Trial limitations, addressing sources of potential bias, imprecision, and, if relevant, multiplicity of analyses | 11-12 |
| Generalisability | 21 | Generalisability (external validity, applicability) of the trial findings | 10-12 |
| Interpretation | 22 | Interpretation consistent with results, balancing benefits and harms, and considering other relevant evidence | 10,12 |
| Other information | | |  |
| Registration | 23 | Registration number and name of trial registry | 5 |
| Protocol | 24 | Where the full trial protocol can be accessed, if available | 5 |
| Funding | 25 | Sources of funding and other support (such as supply of drugs), role of funders | 5 |

*We strongly recommend reading this statement in conjunction with the CONSORT 2010 Explanation and Elaboration for important clarifications on all the items. If relevant, we also recommend reading CONSORT extensions for cluster randomised trials, non-inferiority and equivalence trials, non-pharmacological treatments, herbal interventions, and pragmatic trials. Additional extensions are forthcoming: for those and for up to date references relevant to this checklist, see [www.consort-statement.org](http://www.consort-statement.org).
